# Thermo-sensitive electroactive hydrogel combined with electrical stimulation for repair of spinal cord injury

**DOI:** 10.1186/s12951-021-01031-y

**Published:** 2021-09-23

**Authors:** Wei Liu, Yiqian Luo, Cong Ning, Wenjing Zhang, Qingzheng Zhang, Haifeng Zou, Changfeng Fu

**Affiliations:** 1 Department of Spine Surgery, The First Hospital of Jilin University, 1 Xinmin Street, Changchun, 130021 People’s Republic of China; 2 College of Chemistry, Jilin University, 2699 Qianjin Street, Changchun, 130012 People’s Republic of China; 3 Department of Anesthesia, China-Japan Union Hospital of Jilin University, 126 Xiantai Street, Changchun, 130033 People’s Republic of China

**Keywords:** Spinal cord injury, Polyvaline, Tetraniline, Electroactive hydrogel, Electrical stimulation, Endogenous neurogenesis

## Abstract

The strategy of using a combination of scaffold-based physical and biochemical cues to repair spinal cord injury (SCI) has shown promising results. However, integrating conductivity and neurotrophins into a scaffold that recreates the electrophysiologic and nutritional microenvironment of the spinal cord (SC) remains challenging. In this study we investigated the therapeutic potential of a soft thermo-sensitive polymer electroactive hydrogel (TPEH) loaded with nerve growth factor (NGF) combined with functional electrical stimulation (ES) for the treatment of SCI. The developed hydrogel exhibits outstanding electrical conductance upon ES, with continuous release of NGF for at least 24 days. In cultured nerve cells, TPEH loaded with NGF promoted the neuronal differentiation of neural stem cells and axonal growth, an effect that was potentiated by ES. In a rat model of SCI, TPEH combined with NGF and ES stimulated endogenous neurogenesis and improved motor function. These results indicate that the TPEH scaffold that combines ES and biochemical cues can effectively promote SC tissue repair.

**Supplementary Information:**

The online version contains supplementary material available at 10.1186/s12951-021-01031-y.

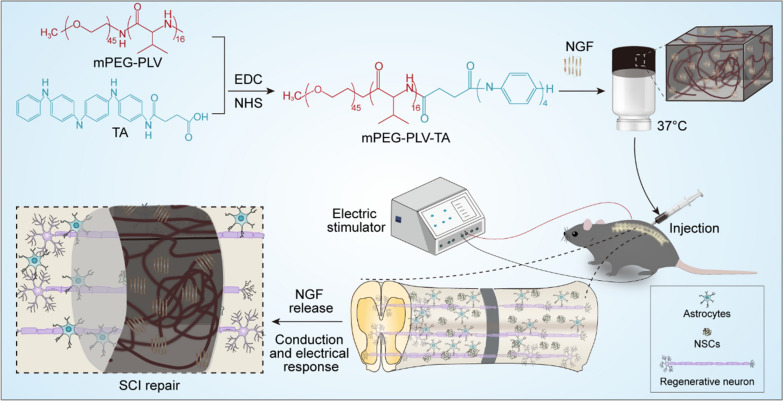

## Introduction

Spinal cord injury (SCI) disrupts the connection between the brain and peripheral organs, leading to sensory motor dysfunction [1]. In addition, SCI can lead to a series of secondary diseases, for example, deep vein thrombosis, muscle spasms, osteoporosis, urinary tract infections, bedsores, chronic pain, and respiratory complications, which are detrimental to the patient's daily activities and even life-threatening [1–3]. Current treatment options for SCI do not yield satisfactory results.

The endogenous bioelectric signals and neurotrophins in the spinal cord (SC) are indispensable in maintaining the physiological activities of neurons, including proliferation, migration, differentiation, and axon growth. [4]. After SCI, the electrical signal transduction is impaired and endogenous neurotrophins are depleted, which hinders tissue repair [5]. Electrical stimulation (ES) is commonly used for the treatment of SCI; in addition to maintaining muscle strength and volume and preventing denervated muscle atrophy, ES regulates the physiologic activities of nerve cells such as proliferation, differentiation, and axon growth [6, 7]. In addition, nerve growth factor (NGF) stimulates axon growth and differentiation of neural stem cells (NSCs) [8, 9]; exogenous NGF can supplement endogenous neuronutrients during SC tissue repair, but multiple administrations are required because of its short half-life in vivo for 4 h [10].

An electroactive neuronutrient bridge can in theory reconnect disrupted neural pathways by continuously release nutrients and responding to external ES to promote endogenous neurogenesis for SCI repair. Hydrogels are an ideal candidate scaffold for this purpose because of their high water content, biocompatibility, 3-dimensional (3D) porous structure, and mechanical properties similar to SC, which meet the requirements of nerve cell adhesion, metabolic substance exchange, and bioactive substance loading for SC regeneration [11, 12]. Hydrogel scaffolds also have electrical conductivity and can simulate the electrophysiologic environment of nerve tissue in combination with exogenous ES. Hydrogels have been used for nerve repair through the delivery of small molecule drugs in a rat brachial plexus injury model [13].

Conductive polymers (CPs) not only have good biocompatibility but also excellent electrical conductivity that can stimulate cell adhesion, proliferation, and differentiation at the polymer–tissue interface to promote tissue growth [14]. These electrically conductive biomaterials can potentially be used to construct neural tissue engineering scaffolds [15]. Polyaniline (PANI) is a CP with excellent physical and chemical properties because of its unique conjugated structure and doping mechanism [16]. However, PANI also has several drawbacks for physiologic applications such as poor flexibility, poor degradability and processing, and low solubility, which can lead to chronic inflammation and pain [17]. Additionally, hydrogels swell under physiologic conditions and ionically or physically loaded PANI could leach out, leading to the decrease of conductivity as well as toxicity.

Poly(amino acid) have excellent properties such as biocompatibility, nontoxicity after degradation, safety, and ease of modification that make them suitable for biomedical applications [18–21]. It is possible that poly(amino acid) can be used to alleviate the shortcomings of CPs for application in neural tissue engineering.

Herein, we prepared an electroactive hydrogel based on poly(amino acid) and the electrically responsive PANI, and combined this with ES to achieve a more potent therapeutic effect in the repair of SC tissue. We grafted tetraniline (TA) onto a previously prepared poly(ethylene glycol)-co-polyvaline (mPEG-PLV) polymer to produce a thermosensitive polymer electroactive hydrogel (TPEH) that was loaded with NGF. The TPEH promoted neuronal differentiation, at the same time inhibiting astrocyte differentiation in vitro, and restored spinal circuitry and locomotor function by stimulating endogenous neurogenesis in a rat SCI model.

## Materials and methods

### Materials

1-Ethyl-3-(3-dimethylaminopropyl)-carbodiimide (EDC), mPEG_2000_-OH, and *N*-phenyl-1,4-phenylenediamine were purchased from Sigma-Aldrich (St. Louis, MO, USA). Triphosgene was obtained from Shanghai Duodian Chemical Co., Ltd. (Shanghai, P. R. China). l-Valine was purchased from GL Biochem, Ltd. (Shanghai, P. R. China). *N*-hydroxysuccinimide (NHS) was purchased from Honeywell Research Chemicals (Charlotte, NC, USA). Dimethyl sulfoxide (DMSO), N,N-dimethylformamide (DMF), tetrahydrofuran (THF), toluene, methylene chloride (CH_2_Cl_2_), chloroform (CHCl_3_), hydrochloric acid (HCl), and other chemicals were purchased from Beijing Chemical Industry Group Co., Ltd. (P. R. China).

### Synthesis and characterization of mPEG-PLV-TA copolymer

#### mPEG-PLV copolymer

mPEG_2000_-NH_2_ was prepared by terminal amination of mPEG_2000_-OH as described in our previous work [22]. Meanwhile, l-Valine *N*-carboxylicanhydride (l-Val NCA) was prepared by mixing triphosgene and l-Val in THF solutio. mPEG-PLV copolymer was obtained from ring-opening polymerization of recrystallized l-Val NCA initiated with mPEG_2000_-NH_2_. In brief, mPEG_2000_-NH_2_ was co-boiled with toluene to remove the water to obtain dry mPEG2000-NH_2_ was added to the DMF solution of l-Val NCA, t. The reaction vessel was stabilized at 25° C through the thermostatic oil bath and stirred for 3 days. The Polymerization product was collected through sedimentation, filtration, and dialysis and freeze-dried under vacuum.

#### Carboxyl-capped aniline tetramer (CTA)

First, tetraaniline (TA) was synthesized as previously reported [23]. Briefly, *N*-pheny-1,4-phenylenediamine (3.68 g, 0.02 mol) was dissolved in a solution of acetone (100 mL), HCl (25 mL), and water (100 mL). In an ice bath, ammonium persulfate (APS, 4.56 g, 0.02 mol) dissolved in hydrochloric acid (50 mL, 1 M) was added dropwise, and the reaction was continued for 3 h to obtain TA. Under nitrogen protection, CTA was obtained by mixing TA and succinic anhydride in CH_2_Cl_2_ solution (5 × the molar weight) at room temperature for 5 h, followed by extraction and filtration. Coarse products were repeatedly washed with distilled water and finally extracted with CH_2_Cl_2_ with a Soxhlet fat extractor to obtain a colorless CTA powder, which was dried under vacuum.

#### mPEG-PLV-TA copolymer

To prepare the mPEG-PLV-TA copolymer, 1.0 mmol mPEG-PLV, 1.0 mmol CTA, 2.0 mmol EDC, and 2.0 mmol NHS were dissolved in a dried glass reactor containing 10.0 mL of DMSO. The mixture was heated to 50 °C for 48 h under a nitrogen atmosphere. After the reaction, the copolymer was precipitated in ether to obtain the crude product. For further refinement, this was dissolved in DMSO and the solution was filtered; the filtrate was dialyzed for 7 days to remove impurities and freeze-dried for 3 days.

### Phase diagram and internal structure

The sol–gel transition behavior of the copolymer in phosphate-buffered saline (PBS) (pH 7.4) was determined with the test tube inversion method. Samples with concentrations ranging from 4.0 to 8.0 wt% were dissolved in PBS and stirred at 4 °C for 12 h. The copolymer solution (0.2 mL) was decanted into a test tube with an inner diameter of 10.0 mm with a gradual increase in temperature. The sol–gel transition temperature was recorded if no flow was observed within 30 s after inverting the test tube. Each data point was the average of three measurements.

To examine the internal structure, hydrogel samples were prepared as described above and lyophilized by freezing in liquid nitrogen for 30 s. The samples were observed by scanning electron microscopy (SEM) (model XL30; Philips, Eindhoven, The Netherlands) at an acceleration voltage of 10 kV.

### In vitro* and *in vivo* hydrogel degradation*

To analyze the degradation properties of the mPEG-PLV-TA hydrogel in vitro, 3.0 mL of PBS without or with elastase (2.0 mg/mL) was slowly added to the surface of hydrogels in cylindrical vials and the samples were placed in a 37 °C incubator with shaking at 70 rpm. After removing the PBS every other day, the vials were weighed, and the recorded values were used to plot the degradation curve.

Wistar rats (weighing ~ 250 g, provided by the Experimental Animal Center of Jilin University, Changchun, China) were used to evaluate gel degradation in vivo. Rats were anesthetized with 2% isoflurane in oxygen (Sevorane; Abbott Spa, Campoverde, Italy), and 0.5 mL of mPEG-PLV-TA PBS solution (6.0 wt%) was injected into the dorsal subcutaneous area using a 21-gauge needle. At 1 h and 7, 14, and 28 days, the skin of rats was carefully cut open to expose the hydrogels, and the hydrogels and surrounding skin were photographed and collected. The skin was fixed overnight with 4% (w/v) paraformaldehyde and embedded in paraffin; the tissue blocks were cut into sections at a thickness of 4 μm that were stained with hematoxylin and eosin (H&E), and inflammation was assessed under a light microscope (model TE2000U; Nikon, Kanagawa, Japan).

### NGF loading and release

The mPEG-PLV-TA hydrogel loaded with NGF was prepared by dissolving 60 mg mPEG-PLV-TA copolymer and 10.0 μg NGF in 1 mL PBS at 4 °C under gentle stirring. The NGF dose was selected based on an earlier study showing that NGF significantly enhanced SCI repair at this concentration [24].

Lysozyme (lys) (Sigma-Aldrich) has a molecular weight and charge similar to those of NGF [25] and is less costly. We used lysozyme to evaluate the in vitro release kinetics of NGF [26, 27]. In order to facilitate detection, the lysozyme was conjugated with fluorescein isothiocyanate (FITC) (Sigma-Aldrich) by combining the two reagents in distilled water at a weight ratio of 5:95 with stirring for 8 h at 4 °C; the solution was then dialyzed and freeze-dried. A mixed solution of mPEG-PLV-TA copolymer and FITC-labeled lysozyme was transferred to cylindrical vials with a diameter of 16 mm that were placed in a water bath at 37 °C until a hydrogel was formed. A 3.0 mL volume of PBS without or with 2.0 mg/mL elastase was slowly added to the vials, which was placed in shaking incubator at 37 °C and 70 rpm. PBS was collected at predetermined time points and fresh buffer was added to the samples. The cumulative release of lysozyme released into PBS was determined by measuring the fluorescence intensity of FITC with a microplate reader (Infinite M200; Tecan, Männedorf, Switzerland); the excitation wavelength was fixed at 495 nm and the emission spectrum was from 510 to 530 nm.

### *Assessment of axon growth *in vitro

PC-12 cells (purchased from BeNa Culture Collection) were grown in Dulbecco’s modified Eagle’s medium (DMEM supplemented with 10% heat-inactivated horse serum and 5% fetal bovine serum (both from Gibco) in a humidified incubator (37 °C, 5% CO_2_), and passaged every other day using 0.25% trypsin at 1:2 dilution. The cells were seeded on sterilized electroactive hydrogel (doped with HCl) coated onto a coverslip with a diameter of 14 mm and treated with poly-l-Lysine at a density of 5 × 10^4^ cells/well. ES was delivered with a waveform Generator (model DG1022U; Rigol, Beijing, China) and the signal was displayed and verified on a digital oscilloscope (model DS1102E; Rigol). A square wave with a frequency of 1 Hz, 5% duty cycle, and electrical potential of 0.1 V was used for stimulation. It’s a cathode stimulation and the pulse peak is alternative current (AC). The ES was performed directly on the surface of the electroactive hydrogel via two microwire platinum electrodes (diameter of 0.5 mm) for 1 h every day. After culturing for 3 days, the cells were fixed with 4% paraformaldehyde at room temperature and stained with 2% FITC in DMSO solution for 10 min, then washed three times with PBS. Neurite morphology was assessed by CLSM; neurite length from the cell body to the most distal tip was measured using ImageJ software (National Institutes of Health, Bethesda, MD, USA).

### NSC culture and differentiation

Newborn Sprague–Dawley rat pups were soaked in 75% ethanol for 0.5 h with only the head exposed, and the SC was removed with sterile forceps on a clean benchtop. The tissue was washed three times with prechilled D-Hank’s solution and quickly transferred to a Petri dish and cut into small pieces using eye scissors that were transferred to a centrifuge tube with a sterile pipette. The medium containing the SC was triturated at a frequency of 10–12 times/min until there was no tissue obstruction and then centrifuged at 1000 rpm for 5 min. The supernatant was removed and sequentially passed through 200- and 500-mesh sieves to remove impurities. NSCs were seeded in a Petri dish at a density of 1 × 10^6^ cells/mL in serum-free medium containing DMEM/F12, 2% B27, 1% penicillin, 20 μg/L basic fibroblast growth factor (bFGF), and 20 μg/L epidermal growth factor (EGF) (all from Gibco), and cultured in an incubator at 37 °C and 5% CO_2_.

NSCs were inoculated in culture plates on the following substrates: slide (control), hydrogel plus ES (Gel + ES), hydrogel plus NGF (Gel + NGF), and hydrogel plus NGF and ES (Gel + NGF + ES). ES parameters are described in “NSC culture and differentiation” section. Immunofluorescence labeling with nestin, class III beta tubulin (Tuj1), and glial fibrillary acidic protein (GFAP) antibodies was performed on days 1 and 7. Images of three random fields were acquired with an inverted microscope (Axio Vert. A1; Leica, Wetzlar, Germany).

### Surgical procedures and treatment of rats

Sprague–Dawley rats were anesthetized with 2% isoflurane in oxygen and the SC was exposed with T10 as the center; the right SC was removed with the posterior midline of the SC as the boundary, forming a 2-mm rectangular defect into which the hydrogels were implanted. Medical gelatin sponge was placed bilaterally in the paravertebral muscular space to absorb bleeding and prevent postoperative adhesion between the connective tissue and dura mater. The muscles were repositioned and sutured and the skin was stapled. To prevent postoperative infection, all rats were intramuscularly injected once daily with ampicillin (150 mg/kg) for 7 days, and the bladder was manually squeezed twice daily until the micturition reflex was restored.

**Scheme 1:**
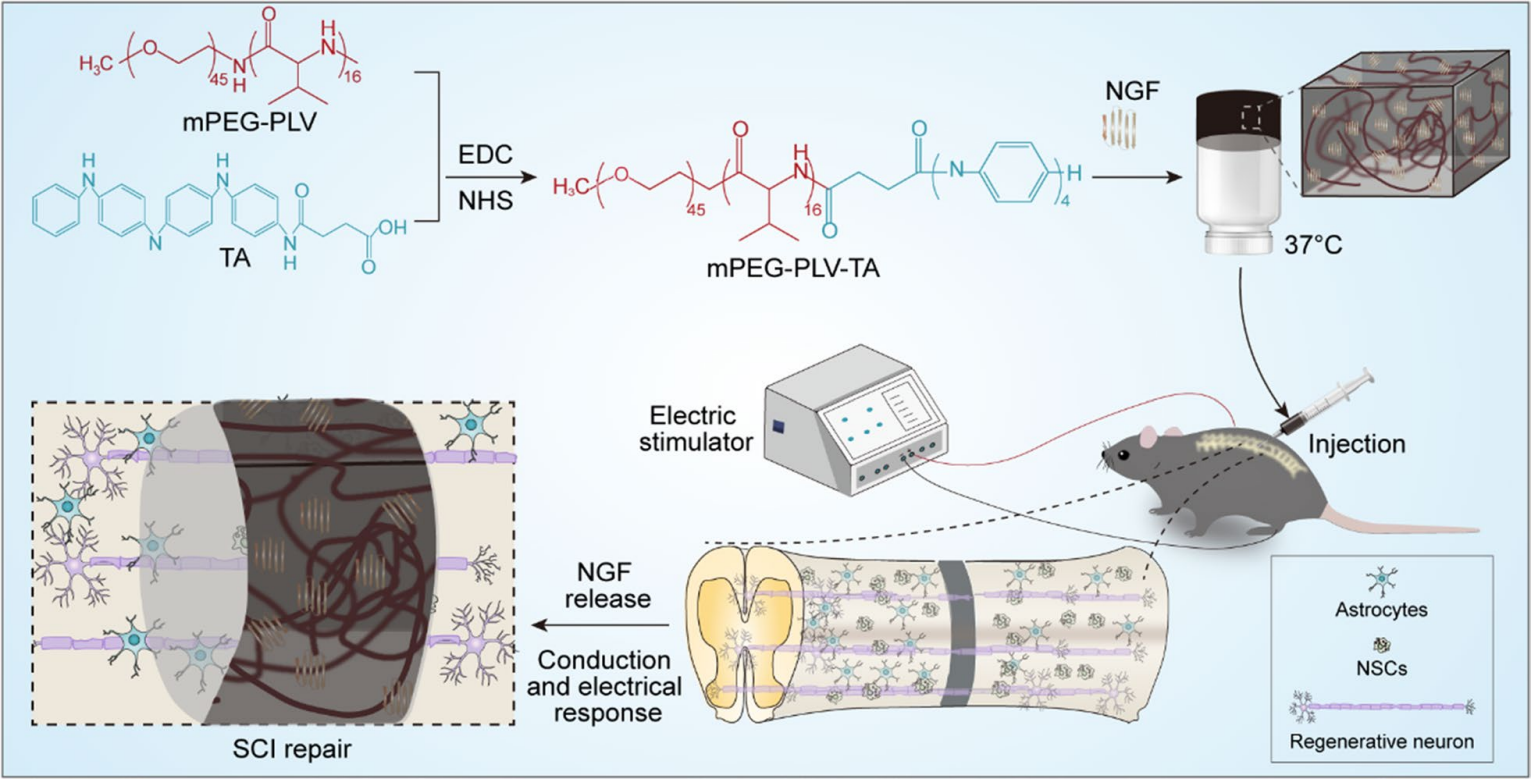
THCP combined with NGF and ES to treat SCI

Transcutaneous ES treatment was performed as follows. Rats were anesthetized with 2% isoflurane in oxygen and placed in a prone position. ES was delivered using a stimulator (model RM6240E; Chengdu Instrument Factory, Chengdu, China) through the skin of rats using an electrode needle. ES (square wave, 10 Hz, 3 mA) was performed seven times (30 min each time) once daily starting on the first postoperative day. The positive electrode was placed on the skin approximately 2 cm to the left of the midline at the posterior spinous process of T9, and the negative electrode was placed on the skin approximately 5 mm below the right knee joint and fibula head (Scheme 1).

## Results and discussion

### Characterization of mPEG-PLV-TA copolymer

**Fig. 1:**
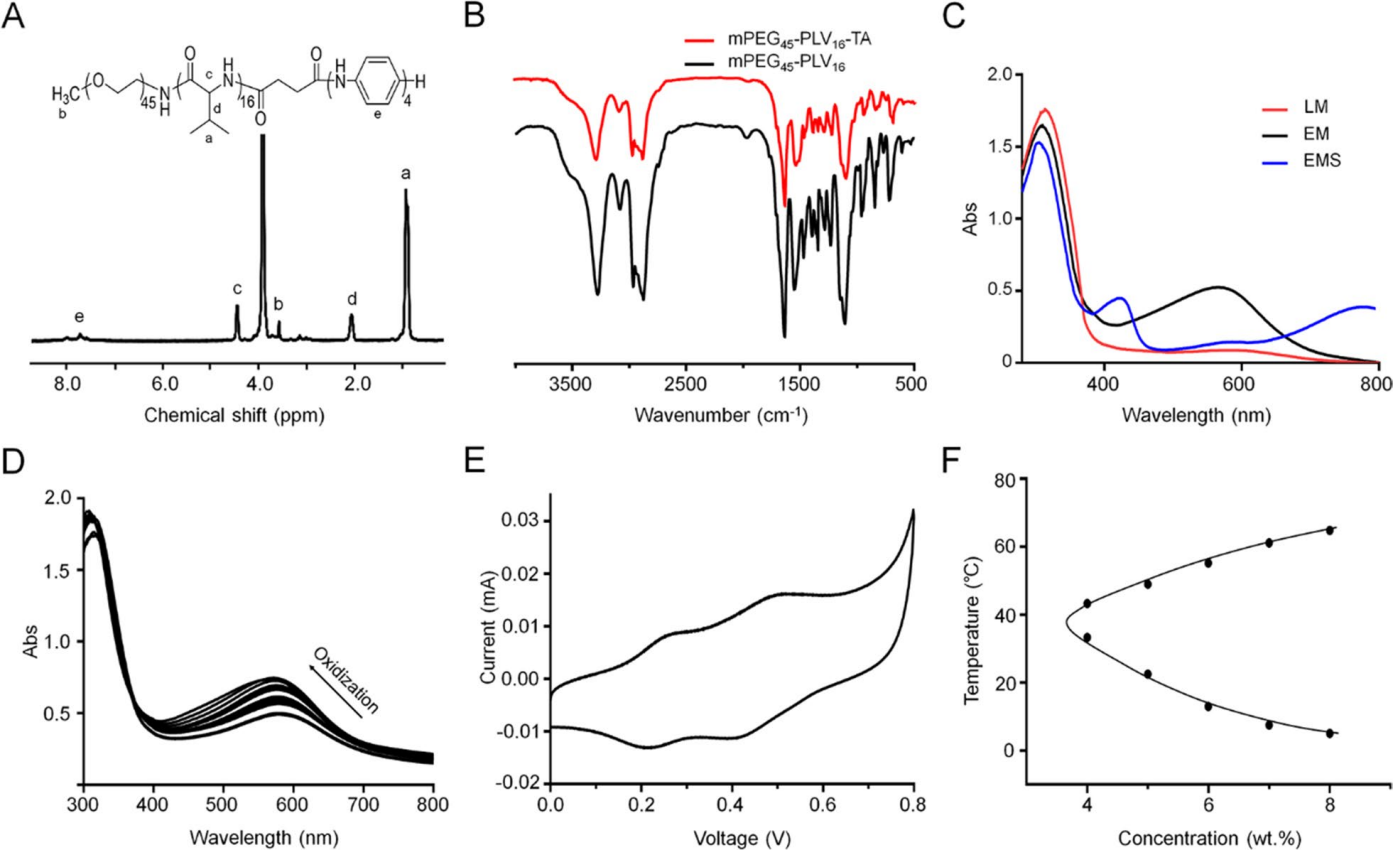
Preparation and characterization of mPEG-PLV-TA copolymers. **A** ^1^H nuclear magnetic resonance spectra of the mPEG-PLV-TA copolymer. **B** Fourier transform infrared spectra of the mPEG-PLV and mPEG-PLV-TA copolymers. **C** UV–vis spectra of the mPEG-PLV-TA copolymer in the LM, EM, PN, and EMS states. **D** UV–vis spectra of the transition of the mPEG-PLV-TA copolymer in DMF from the LM state to the EM state upon oxidation with ammonium persulfate. **E** Cyclic voltamogram of the mPEG-PLV-TA copolymer. **F** Sol–gel phase diagram of the mPEG-PLV-TA copolymer

The electroactive copolymer was synthesized by condensation crosslinking of mPEG-PLV and CTA. To confirm the structure of the electroactive copolymer, we tested ^1^H-nuclear magnetic resonance and Fourier transform infrared spectroscopy. All peaks in the copolymer were clearly assigned (Fig. 1A), demonstrating successful synthesis. Typical CTA absorption peaks were observed at 1603 and 1508 cm^−1^ (Fig. 1B) which corresponded to the stretching vibration of quinone and the benzenoid unit in CTA chain, which indicated that CTA was successfully grafted to mPEG-PLV.

Ultraviolet–visible light (UV–vis) absorption spectra of different oxidation states of the electroactive copolymer are shown in Fig. 1C. There were three oxidation states—i.e., pernigraniline (PNA), emeraldine salt (EMS) and leucoemeraldine (LM) when the copolymer was gradually oxidized, the benzenoid unit was replaced by a benzene ring. In the LM state, the only one absorption peak showed at 310 nm can be related to the π–π* transition of the benzenoid unit. In the EmS state, this absorption peak showed a marked blue shift and a new peak appeared at 575 nm corresponding to the excitonic transition of π–π* from benzenoid to the quinone unit [28]. When the copolymer in the EMS state was doped with HCl (1 M), new characteristic absorption peaks appeared at 430 nm and > 800 nm, reflecting the formation of the EMS state and demonstrating the conductivity of the copolymer after doping [29]. Figure 1D showed the UV–vis spectrum of the gradual oxidation of the mPEG-PLV-TA electroactive copolymer. When ammonium persulfate was gradually added to the DMF solution of the electroactive copolymer, the intensity of the absorption peak at 575 nm gradually increased; after reaching a maximum value, the intensity gradually diminished and showed a blue shift as the degree of oxidation increased.

This redox process was also be observed in the current–voltage curve of the copolymer (Fig. 1E). There are two pairs of redox peaks on the curve, the first pair of more obvious reversible oxidation/reduction peaks, the average peak potential E_1/2_ = (E_pa_ + E_pc_)/2 is 0.25 V, the redox peak corresponds to the reduced state TA and the middle The oxidation–reduction relationship of the oxidation state TA; when the terminal amino group of the aniline tetramer forms an amide bond, it hinders the delocalization ability of the terminal amino group, resulting in the terminal amino group being unable to oxidize to form a quinone ring, so the second pair of redox peaks (corresponding to The redox relationship between the intermediate oxidation state TA and the high oxidation state TA) is not obvious compared to the first pair of redox peaks. The peaks may be due to the presence of part of free TA due to the rupture of the copolymer chains in the acid electrolyte.

From the results of ultraviolet spectrum and cyclic voltammetry spectrum, it can be seen that the aniline tetramer segment in the copolymer maintains good electrical activity in the aqueous solution.

### Gelation ability and internal structure

**Fig. 2:**
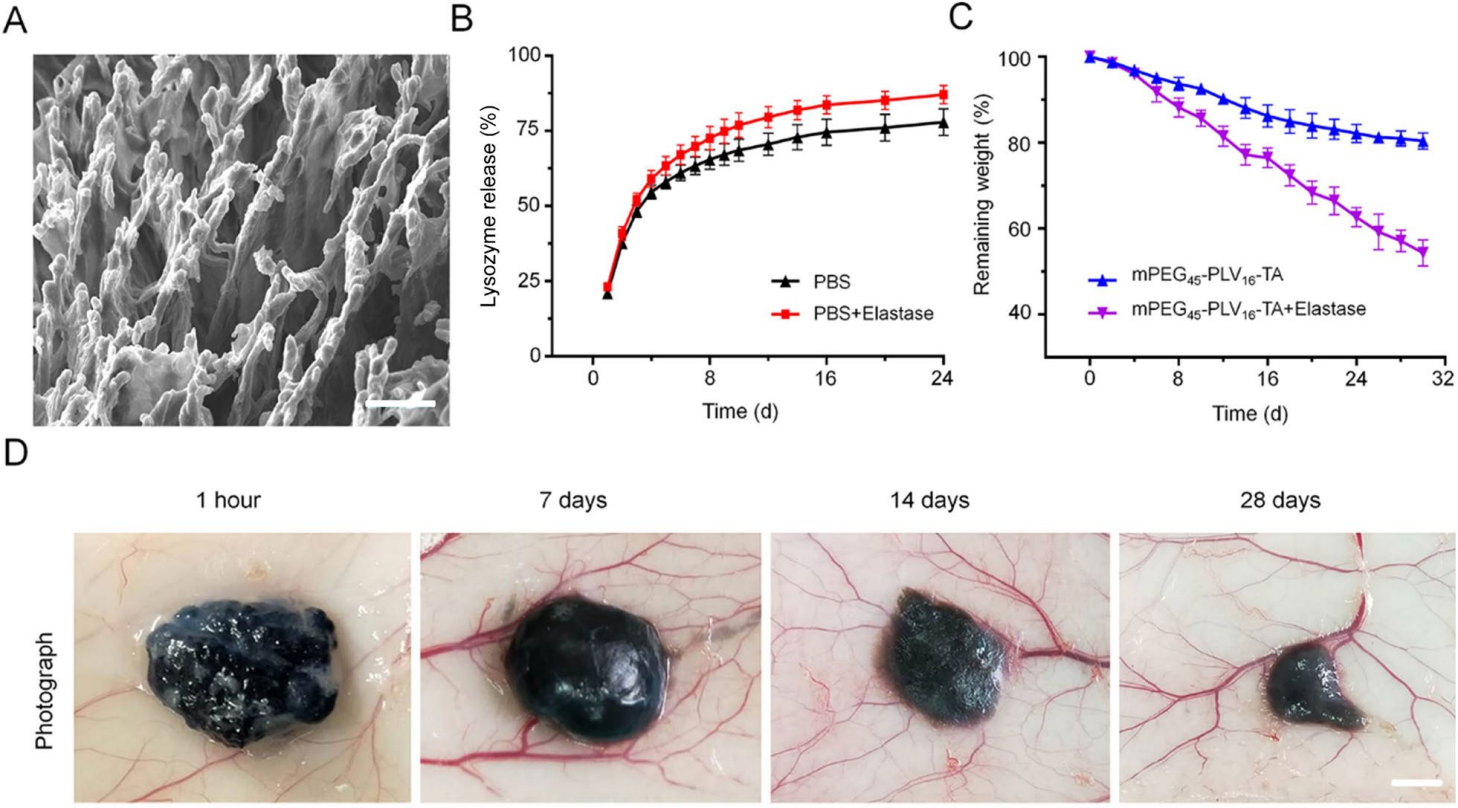
Characterization of the electroactive hydrogel containing growth factors. **A** Microstructure observed by SEM. Scale bar, 50 μm. **B** Release kinetics of NGF in the electroactive hydrogel in PBS without or with elastase. **C** In vitro degradation profiles of the hydrogel in PBS without or with elastase. **D** Biodegradation of the hydrogel and local inflammation in vivo. Scale bar = 200 μm and 50 mm. Data are shown as mean ± SD (n = 3)

Different concentrations of copolymer were dissolved in PBS at 4 °C to obtain homogeneous solutions. The temperature was gradually increased and changes in the state of the solution were examined. As the copolymer concentration increased from 4.0 to 8.0 wt%, a stable gel was formed while the temperature of the sol–gel transition decreased from 33.3 to 5.0 °C (Fig. 1F). Considering the issues of injectability and gelation time during implantation, we selected the 6.0 wt% copolymer solution to prepare electroactive hydrogels for animal experiments. To determine whether the internal structure of the hydrogel was suitable for SC regeneration, we examined the ultrastructure by SEM. The hydrogel had uniform pores with a diameter of 30–70 μm (Fig. 2A), which can serve as channels for tissue fluid, exchange of metabolic substances, drug release, and axon growth.

### NGF release and hydrogel degradation

Long-term nutrient deficiency after SCI impedes the speed and ultimate extent of tissue repair; this problem can potentially be overcome using a scaffold loaded with nutrients, and we therefore investigated whether the hydrogel can efficiently release NGF, which was substituted with lysozyme in this experiment. We loaded 20 mg/mL lysozyme into the electroactive hydrogel and observed the release kinetics [25]. Lysozyme encapsulated in the hydrogel showed burst release (48.28% ± 2.01%) in the first 4 days (Fig. 2B), which may have been due to its leaching out of the hydrogel with water. To simulate the release of lysozyme in the body with hydrogel degradation, elastase (2 mg/L) was added to PBS and release kinetics were monitored [30]. After adding elastase, about 87.02% ± 3.05% of the lysozyme was released from the hydrogel within 24 days, suggesting that the hydrogel can release loaded NGF in vivo over a sufficiently long period of time to allow nerve cell growth after SCI.

We evaluated the in vitro degradation of the hydrogel using samples in PBS alone or PBS containing elastase. Degradation was accelerated in the presence of elastase, with a hydrogel mass loss of > 80.3% ± 1.86% within 30 days (Fig. 2C). These results demonstrate that the hydrogel is degraded at a moderate rate, which can benefit long-term tissue repair.

To further assess the degradation process and biocompatibility, the hydrogel was implanted under the skin of Sprague–Dawley rats. The hydrogel slowly degraded over a period of 4 weeks at a slightly faster rate than in vitro (Fig. 2D). We performed H&E staining of the skin contacting the hydrogel obtained at different time points and found that acute inflammation occurred after the hydrogel was injected and was most prominent on day 7. Over time, the inflammatory response gradually declined and there was no inflammation on day 28 (Additional file 1: Figure S1).

### Biocompatibility and cytotoxicity assessment in vitro

**Fig. 3:**
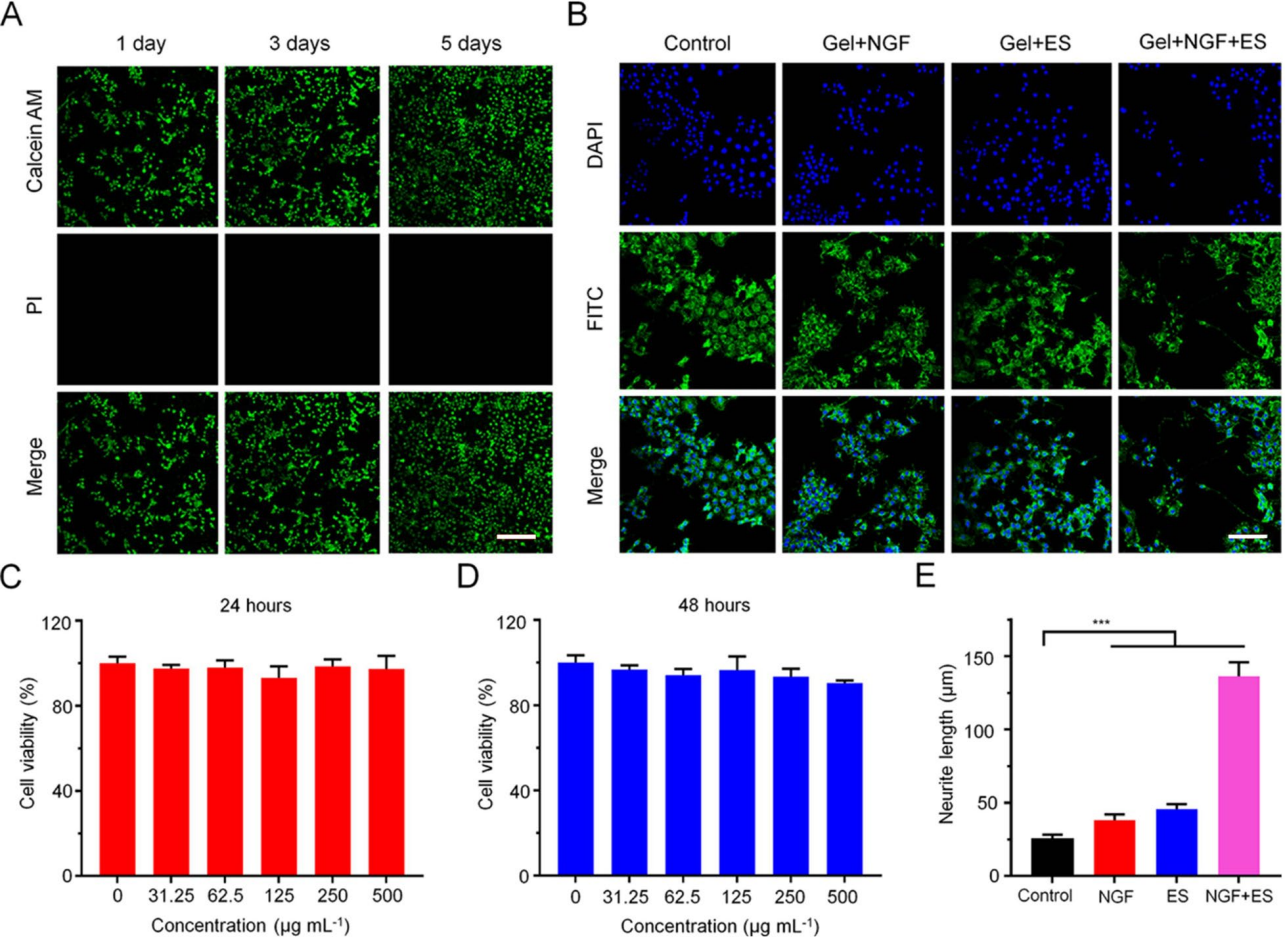
In vitro cytotoxicity of electroactive hydrogels and axon growth in PC12 cells. **A** PC12 cells and hydrogels were cocultured and live–dead staining was performed on days 1, 3, and 5. Scale bar = 100 μm. **B** Effect of hydrogel formulations on axon growth in PC12 cells. Scale bar = 50 μm. **C**, **D** Cell viability after incubation with different concentrations of copolymer for 24 h (**C**) and 48 h (**D**), as determined with the MTT assay. **E** Quantitative analysis of axon length. Data are shown as mean ± SD (n = 3). ***P < 0.001

To evaluate the in vitro cytotoxicity of the electroactive hydrogel, we carried out live–dead cell and MTT assays. PC12 cells were cocultured with hydrogel in mixed calcein-AM and PI dye solution; after 5 days, the cells were proliferating with no evidence of cell death (Fig. 3A). In the MTT assay, when the concentration of the hydrogel suspension increased from 0 to 500 μg/mL, cell viability was in the range of 97.38% ± 6.11% to 100% ± 3.12% after 24 h (Fig. 3C) and 90.40% ± 1.22% to 100% ± 3.48% after 48 h (Fig. 3D). Thus, the electroactive hydrogel has excellent biocompatibility and can be safely used for tissue repair.

### Axon growth and NSC differentiation in vitro

CPs combined with ES can stimulate axon growth to promote nerve repair [31–33]. To investigate whether it can perform this function, the electroactive hydrogel was cocultured with PC12 cells and the length of axons emerging from the cells was analyzed [34]. In the Gel + NGF and Gel + ES groups, only a few cells showed axon-like protrusions; in contrast, most cells in the Gel + NGF + ES showed neurite outgrowth (Fig. 3B). The average neurite length in the Gel + NGF + ES group was 136.30 μm, which was significantly longer than that in the control (25.48 μm), Gel + NGF (38.64 μm), and Gel + ES (45.12 μm) groups (Fig. 3E). These results demonstrate that while the hydrogel by itself has limited ability to stimulate axon growth in nerve cells, the effect can be enhanced by ES and NGF.

**Fig. 4:**
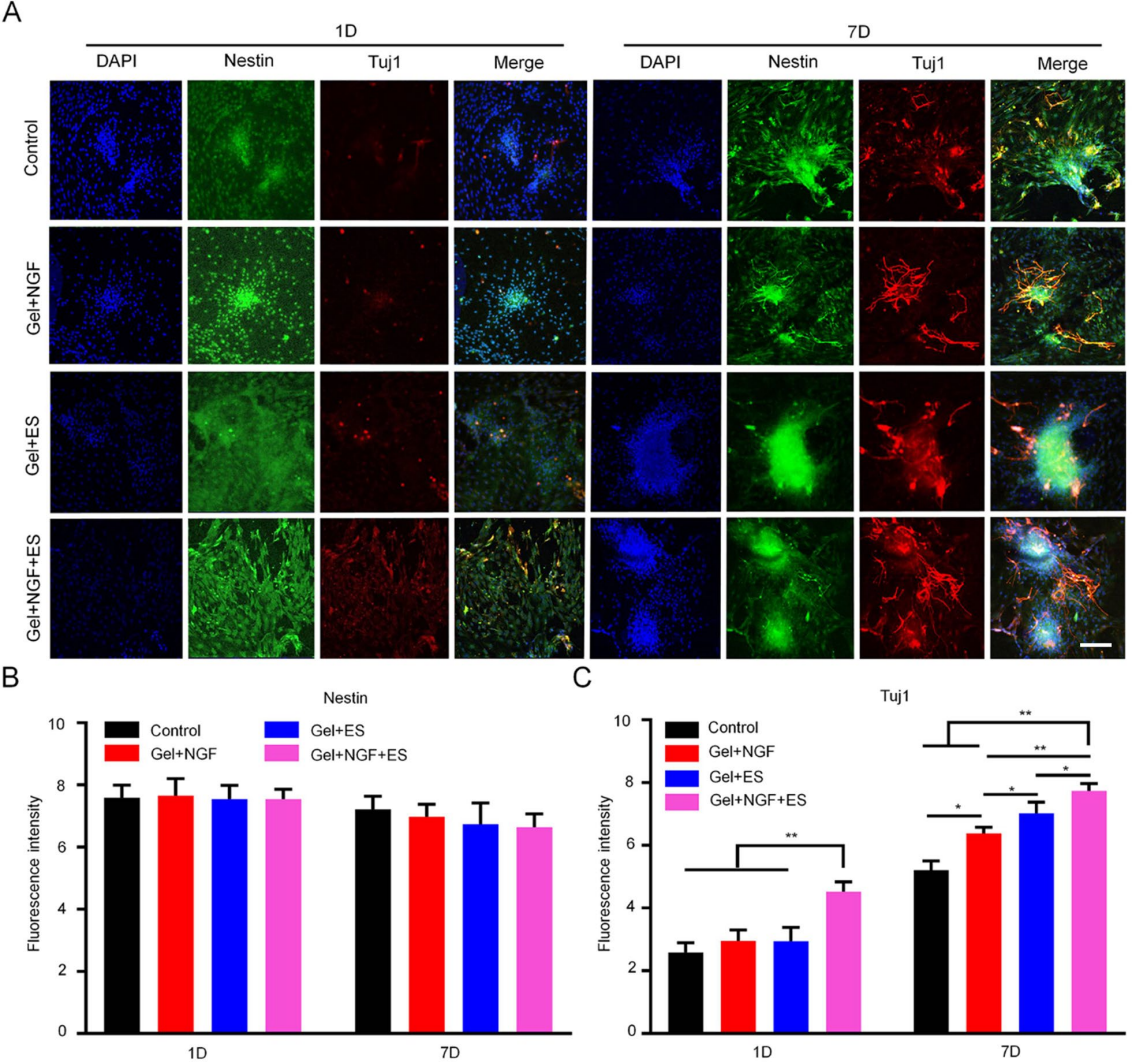
Differentiation of NSCs into neurons in vitro. **A** Analysis of NSC differentiation into neurons by double immunofluorescence labeling of nestin (green) and Tuj1 (red) after 1 and 7 days of treatment. Nuclei were stained with DAPI (blue). Scale bar = 50 μm. **B**, **C** Quantitative analysis of fluorescence intensity of nestin-positive cells (**B**) and Tuj1-positive cells (**C**). Data are shown as mean ± SD. *P < 0.05, **P < 0.01

**Fig. 5:**
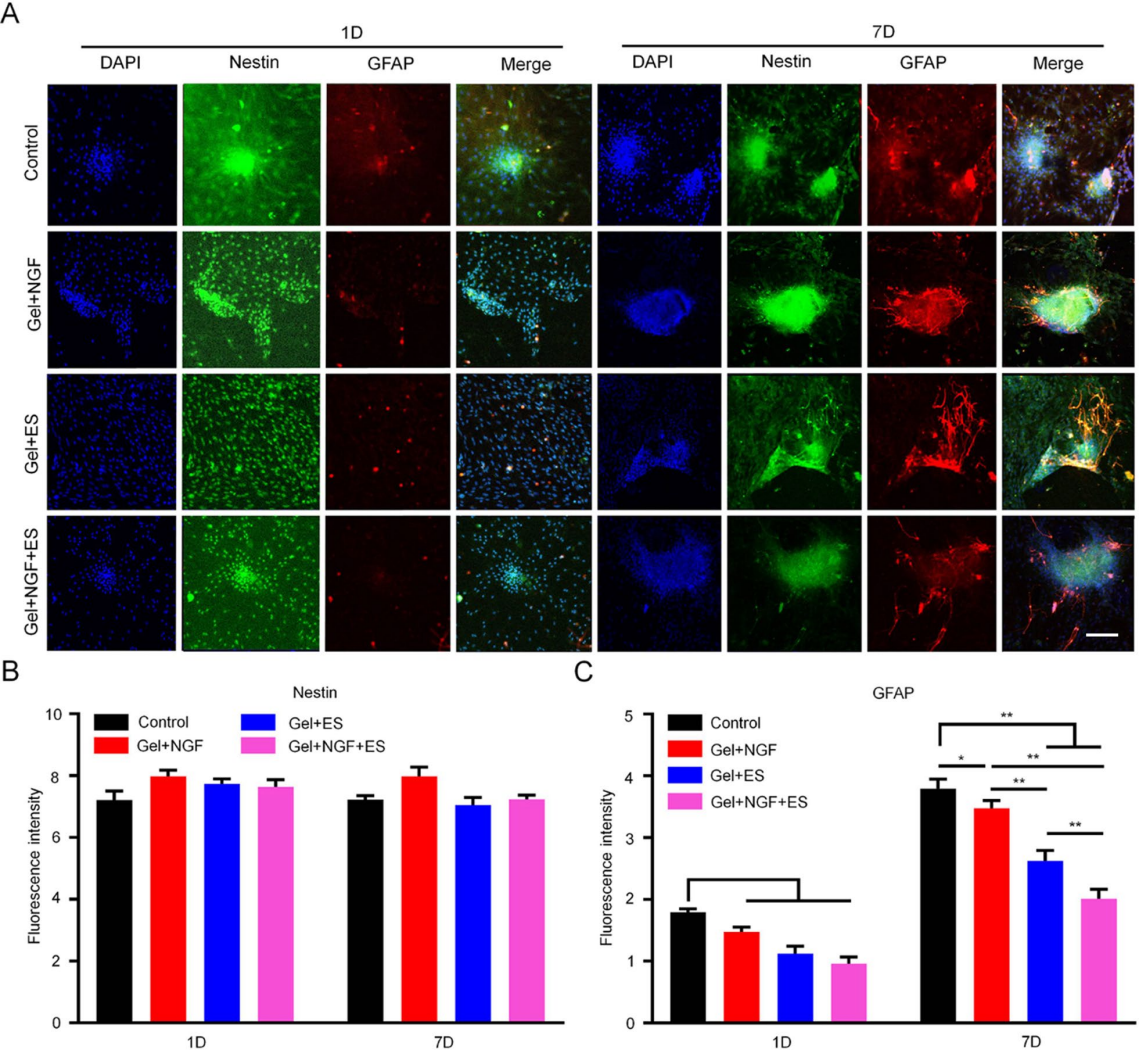
Differentiation of NSCs into astrocytes in vitro. **A** Analysis of NSC differentiation into astrocytes by double immunofluorescence labeling of nestin (green) and GFAP (red) after 1 and 7 days of treatment. Nuclei were stained with DAPI (blue). Scale bar = 50 μm. **B**, **C** Quantitative analysis of fluorescence intensity of nestin-positive cells (B) and GFAP-positive cells (**C**). Data are shown as mean ± SD. *P < 0.05, **P < 0.01

In addition to promoting axon growth, replacing dead neurons in the SC is an important strategy for restoring motor function. The softer hydrogel helps neural stem cells differentiate into neurons [35]. To verify whether our hydrogel can promote a large-scale differentiation of NSCs into neurons, NSCs were cocultured with the hydrogel and immunolabeling was performed to assess their differentiation status after 1 and 7 days. We found that the hydrogel induced the differentiation of NSCs into neurons and that the effect was potentiated by ES. This was accompanied by changes in cell morphology: neurites of a single neuron extended in all directions, forming an extensive network with other neurons (Fig. 4A). During this process, there were no changes in the expression level of nestin, a marker of NSCs, in any treatment groups (Figs. 4B and 5B). However, there was a significant increase in Tuj1 expression in all groups from day 1 to 7; the average fluorescence intensity on day 7 was 8.73 in the Gel + NGF + ES group, which was significantly higher than that in the control (5.21), Gel + NGF (6.37), and Gel + ES (7.02) groups (Fig. 4C). In contrast, the hydrogel had an inhibitory effect on NSC differentiation into astrocytes (Fig. 5A): the average fluorescence intensity of GFAP was lower in the Gel + NGF + ES group (4.01) on day 7 than in the control (7.58), Gel + NGF (6.95), and Gel + ES (5.24) groups (Fig. 5C). These results suggest that the hydrogel inhibits astrocyte proliferation and may prevent the formation of glial scars.

### Recovery of motor function and nerve conduction

**Fig. 6:**
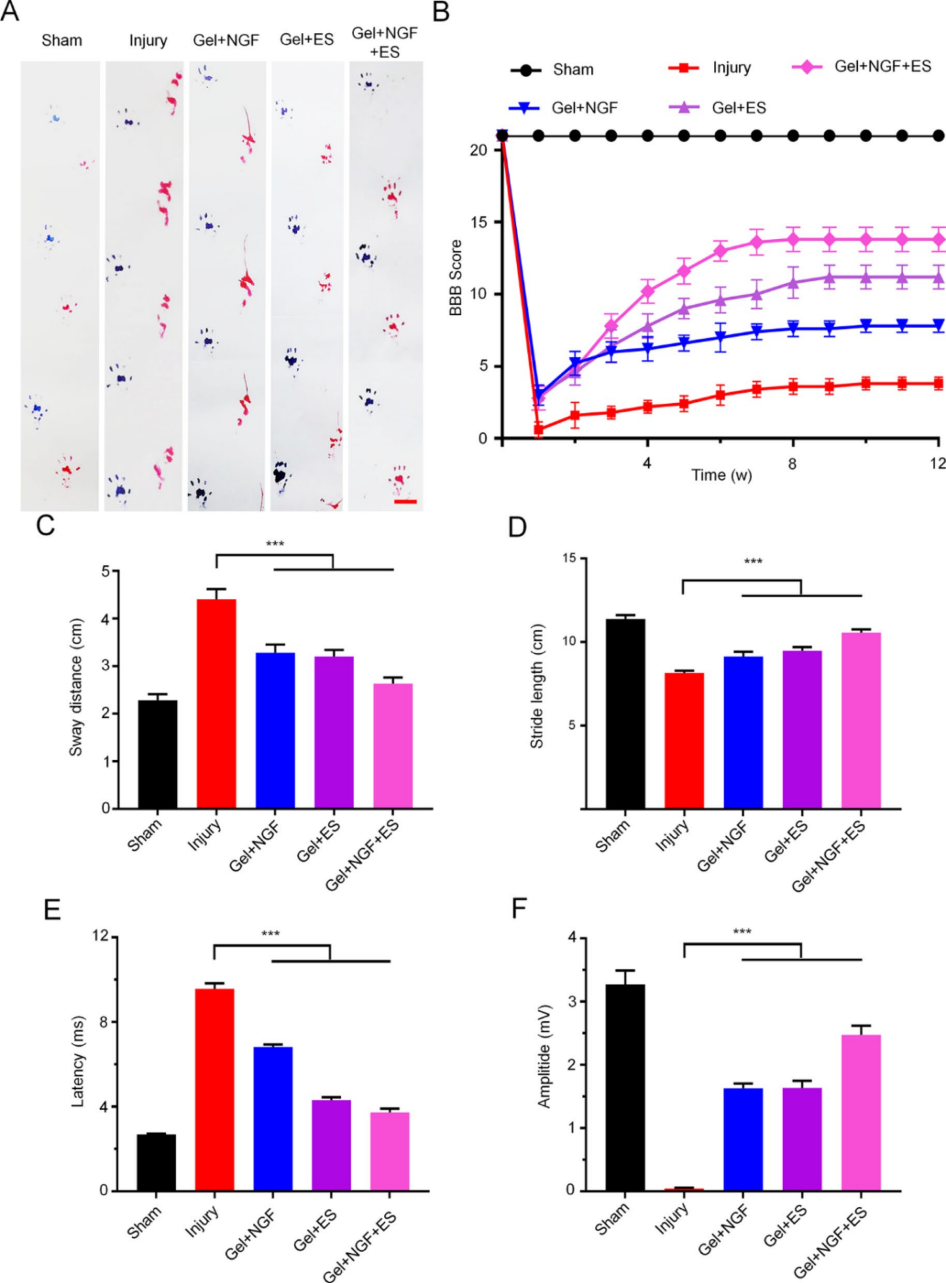
Functional recovery post SCI. **A** Representative footprint patterns of different groups at 12 weeks after SCI. Scale bar = 2.5 cm. **B** BBB score of rats at 12 weeks after SCI. (C, D) Semiquantitative analysis of stride length (**C**) and sway distance (**D**) in the footprint test. **E**, **F** MEP latency (**E**) and amplitude (**F**). Data are shown as mean ± SD (n = 3). ***P < 0.001

The restoration of motor function is the most important index for gauging SC repair. For the purpose of investigate the ability of the hydrogel to promote functional recovery, the footprint test was conducted and BBB score was evaluated in rats with SCI. At postoperative week 12, the sham group showed a strong and stable normal gait in the footprint analysis, whereas injured rats showed a decreased stride length and increased sway distance with severe toe dragging (Fig. 6A). The treatment groups showed varying degrees of gait recovery: rats in the Gel + NGF + ES group had a stride length (10.55 ± 0.21 cm) that was closer to that of the sham group (11.38 ± 0.22 cm) than the Gel + NGF (9.13 ± 0.29 cm) and Gel + ES (9.48 ± 0.21 cm) groups, indicating that the recovery of motor function was greatest with the hydrogel loaded with NGF combined with ES (Fig. 6C). The same trend was observed for sway distance (2.63 ± 0.13 cm in the Gel + NGF + ES group vs 2.28 ± 0.13 cm in the sham group) (Fig. 6D). Abnormal gait may be caused by decreased muscle tone, cerebellar dysfunction, impaired peripheral nerve function, or musculoskeletal abnormalities [36]. In this study, the degree of gait recovery was different, which could be due to the different degrees of regeneration and conduction of nerve cells in the gray and white matter, respectively; alternatively, inconsistent muscle tension caused by atrophic lower limb muscles may account for the observed differences.

To monitor the recovery of motor function of rats with SCI, we determined the BBB score in multiple different time points. The right hind limb of SCI model rats was paralyzed immediately after the operation, and the BBB score was close to 0 (Fig. 6B); even after 12 weeks, the score did not exceed 4 points, indicating that there was very little recovery after injury. In the treatment groups, the BBB score gradually increased over time; starting from week 4 after surgery, the fraction of Gel + NGF, Gel + ES, and Gel + NGF + ES groups are significantly higher scores than the SCI model group and after 12 weeks, the BBB score of the Gel + NGF + ES group (13.8 ± 0.84) was higher than those of the other treatment groups.

We also evaluated the recovery of nerve conduction by measuring MEP. The amplitude of the MEP was reduced by SCI while the latency was increased, and there was no improvement in these parameters over time (Fig. 6E, F). After 12 weeks, the amplitude was increased in all treatment groups and the latency was shortened. The amplitude of the MEP was obviously higher in the Gel + NGF + ES group (2.47 ± 0.14 mV) than in the Gel + NGF (1.62 ± 0.08 mV) and Gel + ES (1.63 ± 0.11 mV) groups. Similarly, the latency was shorter in the Gel + NGF + ES group (3.73 ± 0.18 ms) than in the Gel + NGF (6.81 ± 0.13 ms) and Gel + ES (4.3 ± 0.1 ms) groups. Taken together, these results demonstrate that the NGF-loaded electroactive hydrogel combined with ES promoted the recovery of motor function in rats after SCI.

### Tissue repair in the SC

**Fig. 7:**
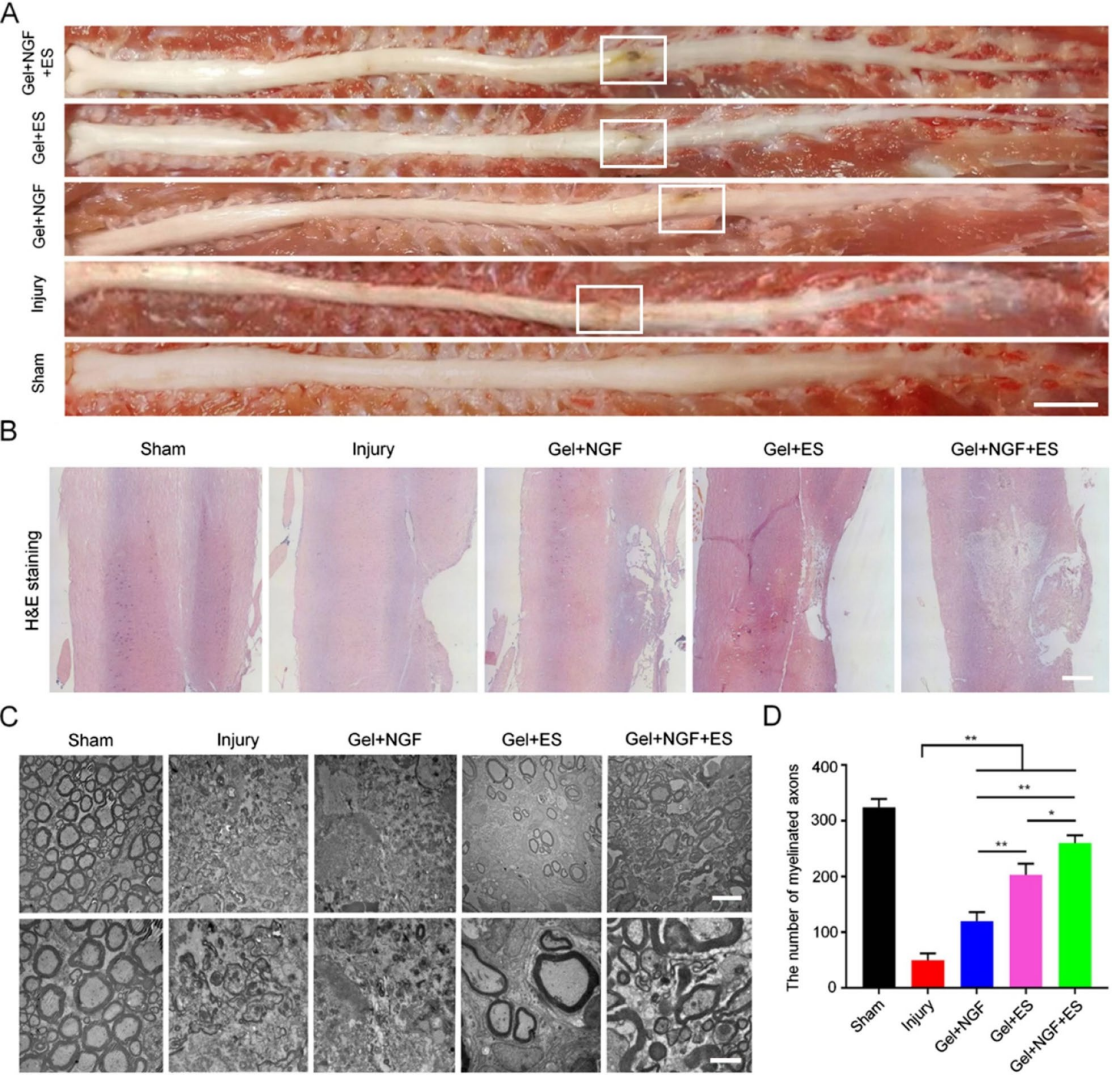
SC tissue repair. **A** Gross morphology of the SC in different groups. Scale bar, 1 cm. **B** Histologic analysis of SC tissue by H&E staining. Scale bar = 1 mm. **C** TEM analysis of SC ultrastructure in different groups. Scale bar = 50 μm. **D** Quantitative analysis of the number of axons at lesion sites. Data are shown as mean ± SD (n = 3). **P < 0.01, ***P < 0.001

We carried out histologic and ultrastructural analyses to confirm the repair of SC tissue. Rats were sacrificed 12 weeks post surgery and the SC was completely dissected. H&E staining of tissue sections revealed that there was no new tissue growth in the SCI model group (Fig. 7A). However, the Gel + NGF + ES group had smallest cavity area corresponding to the site of injury and more new tissue than the other treatment groups (Fig. 7B). A closer examination of SC ultrastructure by TEM indicated that compared to the sham group, SCI model rats had scar tissue hyperplasia and demyelination, with no obvious myelin sheath or axon regeneration, whereas myelinated axons were observed in the Gel + NGF, Gel + ES, and Gel + NGF + ES groups (Fig. 7C, D). These results demonstrate that the hydrogel with NGF and ES can promote nerve cell growth and SC tissue recovery.

### Differentiation of NSCs into neurons and inhibition of glial scar formation in vivo

**Fig. 8:**
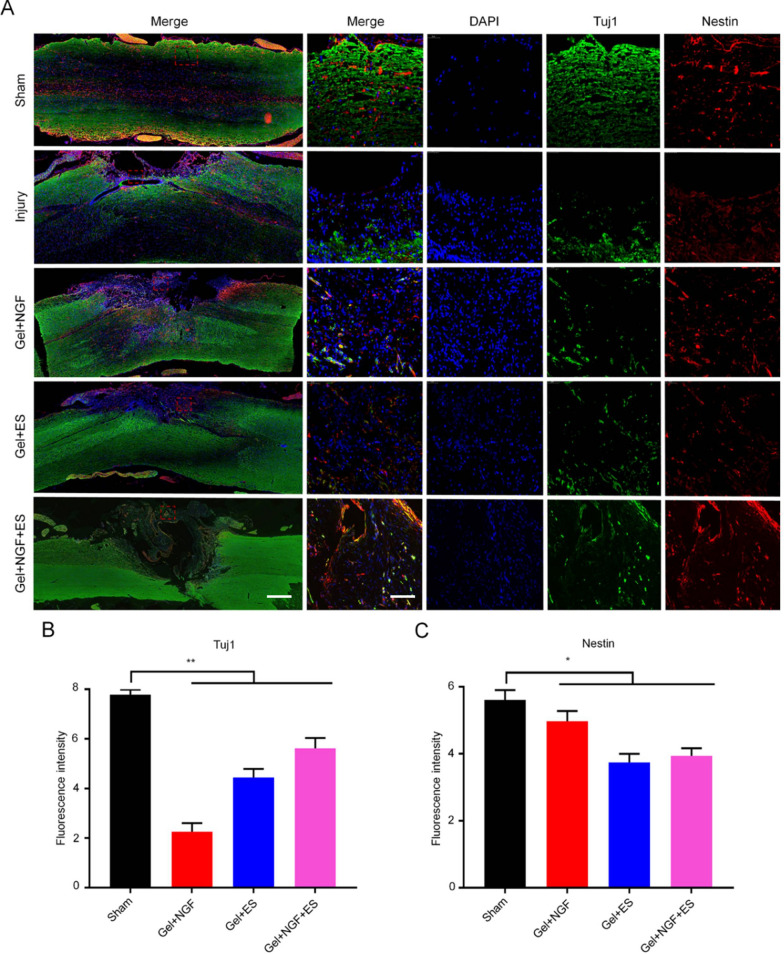
Investigation on NSCs differentiate into neurons in vivo*.* **A** Analysis of NSC differentiation into neurons by double immunofluorescence labeling of nestin (red) and Tuj1 (green) after 12 weeks of treatment. Nuclei were stained with DAPI (blue). Scale bar, 50 μm. **B**, **C** Quantitative analysis of fluorescence intensity of nestin-positive cells (**B**) and GFAP-positive cells (**C**). Data are shown as mean ± SD (n = 5). *P < 0.05, **P < 0.001, ***P < 0.001

To determine whether the electroactive hydrogel loaded NGF combined with ES can facilitate the differentiation of NSCs into neurons in vivo, we performed double immunofluorescence labeling of SC tissue specimens with antibodies against nestin and Tuj1. There were no newborn neurons (Tuj1-positive cells) in the SCI model group, but they were observed in rats treated with Gel + NGF, Gel + ES, and Gel + NGF + ES (Fig. 8A). Notably, nestin and Tuj1 showed different degrees of expression and colocalization in areas of new tissue, suggesting that NSCs migrated from intact parts of the SC to the site of injury before differentiating into neurons. A semiquantitative analysis illustrated that the rate of neurogenesis in the Gel + NGF + ES group was 1.2 and 3.1 times higher than that in Gel + NGF and Gel + ES groups, respectively (Fig. 8B, C). Although it seems that the damage area of Gel + NGF + ES group is larger in terms of area, the damaged area is basically full of newly generated neuron cells, which is beneficial to the further repair of nerves. In the other treatment groups and injury groups, since the differentiation of neural stem cells into neurons was not stimulated, the cells in the damaged area were mainly other non-neuronal cells. This is not very helpful for the further repair of nerves.

**Fig. 9:**
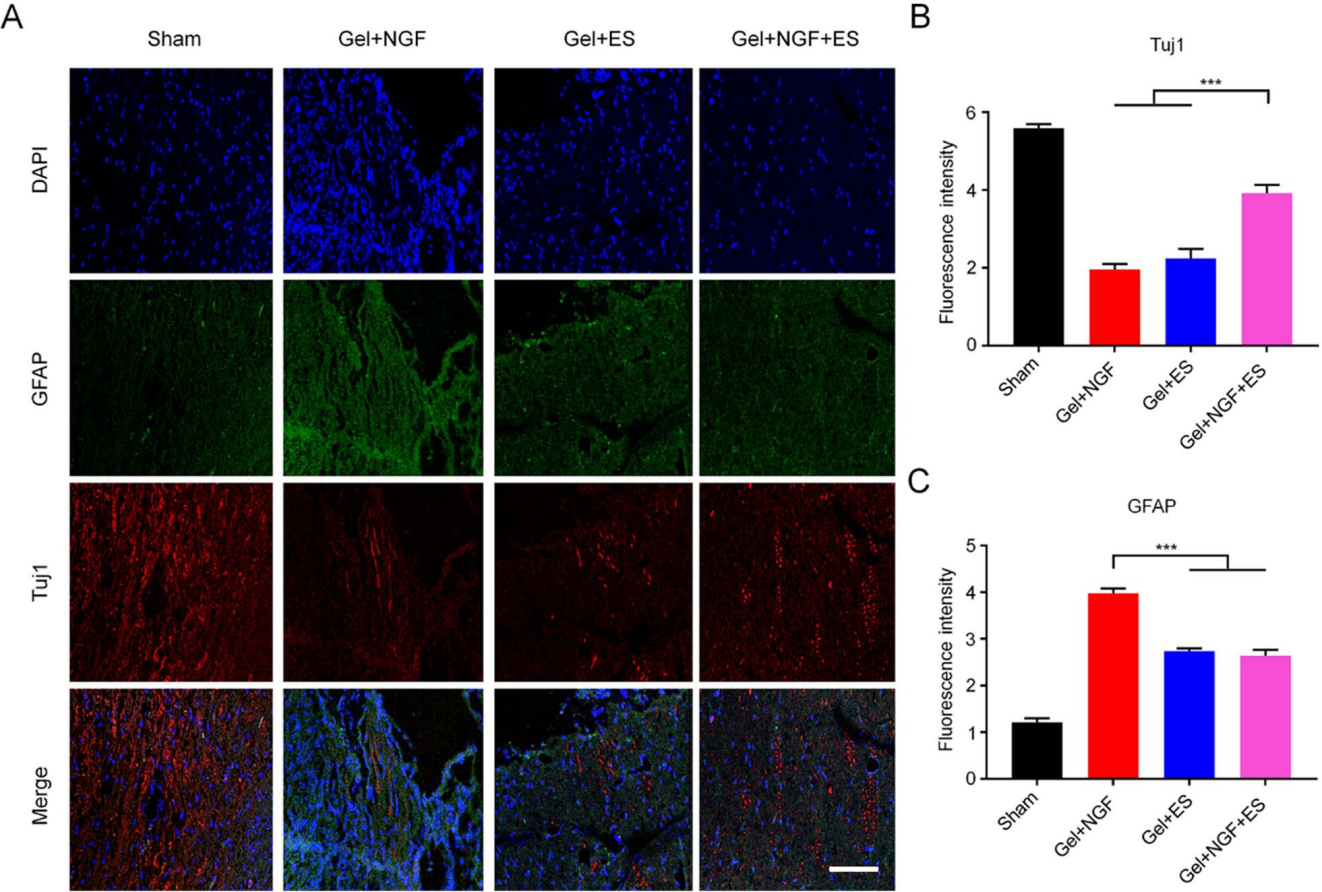
Axon growth and inhibition of glial scar. **A** Analysis of axon growth and inhibition of glial scar formation by double immunofluorescence labeling of GFAP (green) and Tuj1 (red) after 12 weeks of treatment. Nuclei were stained with DAPI (blue). Scale bar, 50 μm. **B**, **C** Quantitative analysis of fluorescence intensity of Tuj1-positive cells (**B**) and GFAP-positive cells (**C**). Data are shown as mean ± SD (n = 5). *P < 0.05, **P < 0.001, ***P < 0.001

Astrocytes are the dominant type of glial cell in the central nervous system and their continuous activation and hyperproliferation after SCI can lead to the formation of glial scars, which can prevent SC regeneration. Double immunolabeling of GFAP and Tuj1 in SC tissue sections proved that GFAP expression was higher in SCI model rats than in sham group (Fig. 9A, C) and higher in the Gel + NGF group than in the Gel + ES and Gel + NGF + ES groups. This may be related to the fact that ES inhibits glial scar formation. In addition to being expressed by immature neurons, Tuj1 is also an axon marker. Tuj1 expression was observed in all treatment groups, and the level in the Gel + NGF + ES group was significantly higher than that in the Gel + NGF and Gel + ES groups (Fig. 9A, B). Thus, the electroactive hydrogel loaded with NGF can promote axon regeneration in the injured SC, with ES enhancing this effect.

## Conclusion

In summary, we prepared an electroactive hydrogel scaffold by grafting conductive TA onto poly(l-valine). The hydrogel was porous and showed good biocompatibility, biodegradability, and conductivity, and effectively stimulated SC tissue repair through continuous slow release of NGF and in response to external ES. These features induced the differentiation of NSCs into neurons in the injured SC while inhibiting the proliferation of astrocytes. Implantation of the hydrogel in SCI model rats stimulated endogenous neurogenesis and promoted the restoration of motor function. These results provide evidence that the electroactive hydrogel loaded with NGF and combined with ES has clinical potential for the treatment of SCI.

## Supplementary Information

**Additional file 1: Figure S1.** Biocompatibility of electroactive hydrogel. Scale bar = 200 μm.

## Data Availability

All relevant data supporting the findings of this study are either included within the article and its additional files or available upon request from the corresponding author.

## Abbreviations

BBB: Basso, Beattie, and Bresnahan
CLSM: Confocal laser scanning microscopy
CP: Conductive polymer
CTA: Carboxyl-capped aniline tetramer
DAPI: 4′,6-Diamidino-2-phenylindole
DMEM: Dulbecco’s modified Eagle’s medium
DMF: *N*,*N*-Dimethylformamide
DMSO: Dimethyl sulfoxide
EDC: 1-Ethyl-3-(3-dimethylaminopropyl)-carbodiimide
EMS: Emeraldine salt
ES: Electrical stimulation
FITC: Fluorescein isothiocyanate
GFAP: Glial fibrillary acidic protein
H&E: Hematoxylin and eosin
LM: Leucoemeraldine
L-Val NCA: L-valine-*N*-carboxylicanhydride
MEP: Motor evoked potential
mPEG: Poly(ethylene glycol) methyl ether
mPEG-PLV: Poly(ethylene glycol)-co-polyvaline
MTT: 3-(4,5-Dimethylthiazol-2-yl)-2,5-diphenyltetrazolium bromide
NGF: Nerve growth factor
NHS: N-hydroxysuccinimide
NSC: Neural stem cell
PANI: Polyaniline
PBS: Phosphate-buffered saline
PI: Proidium iodide
PN: Pernigraniline state
SCI: Spinal cord injury
SEM: Scanning electron microscopy
TA: Tetraniline
TEM: Transmission electron microscopy
THF: Tetrahydrofuran
TPEH: Thermosensitive polymer electroactive hydrogel
Tuj1: Class III beta tubulin
UV–vis: Ultraviolet–visible light
