# Supplementary material for: Thermo-sensitive electroactive hydrogel combined with electrical stimulation for repair of spinal cord injury
Source: J Nanobiotechnology. 2021 Sep 23;19:286. doi: 10.1186/s12951-021-01031-y (PMC8461877; doi:10.1186/s12951-021-01031-y)
Supplement: Supplementary file 1 — Additional file 1: Figure S1. Biocompatibility of electroactive hydrogel. Scale bar = 200 μm. [file 12951_2021_1031_MOESM1_ESM.docx]

**Additional file**

**Thermo-sensitive electroactive hydrogel combined with electrical stimulation for repair of spinal cord injury**

Wei Liu^a,b^, Yiqian Luo^a^, Cong Ning^a^, Wenjing Zhang^c^, Qingzheng Zhang^a^, Haifeng Zou^a,^*, Changfeng Fu^b,^*

^a^ *Department of Spine Surgery, The First Hospital of Jilin University, 1 Xinmin Street, Changchun 130021, P. R. China*

^b^ *College of Chemistry, Jilin University, 2699 Qianjin Street, Changchun 130012, P. R. China*

^c^ *Department of Anesthesia, China-Japan Union Hospital of Jilin University, 126 Xiantai Street, Changchun, 130033, P. R. China*

*Corresponding authors.

*E-mail addresses:* fucf@jlu.edu.cn (C. Fu), zouhf@jlu.edu.cn (H. Zou).

**1. Experimental**

*1.1. Characterizations*

Nuclear magnetic resonance (NMR) spectra were recorded on AV-300 spectrometer (Bruker) at room temperature in trifluoroacetic acid-*d* (TFA-*d*) or D_2_O. Scanning electron microscopy (SEM) was texted by XL30 SEM (Philips) at an acceleration voltage of 10 kV. Fourier-transform infrared (FT−IR) spectra were recorded on a Bio-Rad Win-IR instrument using potassium bromide method. Gel filtration chromatography (GFC) measurements were conducted on a waters GFC system (Waters Ultrapydrogel Linear column, 1515 HPLC pump with 2414 Refractive Index detector) using phosphate buffer (0.2 M, pH 7.4) as eluent (flow rate: 0.8 mL min−1, 35°C, and polyethylene glycol as standards). The change of particle size was determined by dynamic laser scattering (DLS) on Zetasizer Pro (Malvern Instrument, UK).

*1.2. Cytotoxicity assessment*

The cytotoxicity of the hydrogel was assessed by live–dead staining and the 3-(4,5-dimethylthiazol-2-yl)-2,5-diphenyltetrazolium bromide (MTT) assay. Briefly, pheochromocytoma (PC-12) cells were seeded on the electroactive hydrogel in a 24-well plate and cultured for 1, 3, or 5 days. The culture medium was replaced with a solution of 2 μM calcein AM and 4.5 μM proidium iodide (PI) (Calcein-AM/PI Double Stain Kit; Yeasen, Shanghai, China) in PBS, followed by incubation for 30 min. The cells were observed by confocal laser scanning microscopy (CLSM). Living cells were stained green by calcein AM and dead cells were stained red by PI [1].

For the MTT assay, PC-12 cells were seeded at a density of 8×10^3^ cells/well in a 96-well plate in 180.0 µL Dulbecco’s modified Eagle’s medium (DMEM; Gibco, Grand Island, NY, USA). After incubation for 24 h, 20.0 µL of copolymer solution at different concentrations (31.3–500.0 µg/mL) was added. The cells were incubated for another 24 or 48 h, and 20.0 μL of PBS containing MTT (0.05 mg/mL) was added, followed by incubation for 4 h. The medium was replaced with 160.0 µL of dimethyl sulfoxide (DMSO). The absorbance of the solution was measured at 490 nm on a microplate reader (model 680; Bio-Rad, Hercules, CA, USA). Cell viability (%) was calculated using equation (1):

Cell viability = *A*_sample_ / *A*_control_ × 100 (1)

where *A*_sample_ and *A*_control_ are the absorbance values of the sample and control wells, respectively.

*1.3. Functional recovery assessment*

Functional recovery after SCI was evaluated starting at 12 weeks after the operation with the Basso, Beattie, and Bresnahan (BBB) score, footprint test, and by electrophysiologic recording. The recovery of right lower limb function was evaluated weekly using the BBB score [2]. The footprint test was conducted as follows. The plantar surface of both hind limbs of each rat was colored blue (left) and red (right) with nontoxic ink. The rat was allowed to run toward a dark tunnel on a white paper (21×59.4 cm^2^). Stride length (distance between the centers of adjacent ipsilateral footprints) and sway distance (perpendicular distance between the centers of left and right hind limbs) were measured, and the average of five steps in each animal was used for statistical comparisons.

Motor evoked potential (MEP) was conducted 12 weeks postoperatively. Rats were anesthetized with 2% isoflurane in oxygen and placed on a stereotactic apparatus. The skin of the posterior part of the skull was sterilized with iodophor and cut and the periosteum was removed. A dental drill was used to create a blind hole in the skull above the central posterior gyrus of the right cortex (3 mm behind the coronal suture and 1 mm beside the sagittal suture). The skin of the left lower limb was cut and the sciatic nerve was exposed. The positive electrode was placed in the cerebral cortex; the negative electrode was placed under the scalp; the recording electrode was placed in the sciatic nerve; and the reference electrode was placed on the skin of the hip. Stimulation parameters were as follows: crude voltage intensity, 3 V; wave width, 1 ms, frequency, 10 Hz, gain, 20×, and filter, 300 Hz.

*1.4. Tissue dissection and H&E staining*

Rats were deeply anaesthetized by intraperitoneal injection of pentobarbital sodium (50 mg/kg). Conventional perfusion fixation with 4% (*w/v*) paraformaldehyde was carried out and the full length of the SC was exposed and photographed. T9–T11 was dissected and fixed overnight with 4% (w/v) paraformaldehyde, dehydrated in gradient ethanol, and embedded in paraffin. The tissue blocks were cut into sections at a thickness of 4 μm that were stained with H&E for histologic analysis.

*1.5. Analysis of spinal ultrastructure*

At 12 weeks after surgery, three rats from each group were randomly selected and sacrificed by anesthetic overdose. After cardiac perfusion with a mixture of 4% paraformaldehyde and 2% glutaraldehyde (w/v), the SC was dissected and then fixed in a mixture of 4% paraformaldehyde and 2% glutaraldehyde at 4 °C for 6 h. The regenerated tissue was immersed in 1% osmium tetroxide for 2 h, then washed several times with 0.075 M PBS, dehydrated in gradient ethanol and acetone, and embedded in epoxy resin. Semithin sections were cut at a thickness of 1 μm, stained with hematoxylin, and observed under a light microscope to select areas suitable for ultrathin sectioning; the latter sections were stained with uranyl acetate and lead citrate and SC ultrastructure was examined by transmission electron microscopy (TEM, model JEM-1011; JEOL, Toyko, Japan).

*1.6. Immunofluorescence analysis*

Laminectomy was performed after transcardial perfusion. T9–T11 of the SC was microdissected and postfixed by immersion in ice-cold 4% paraformaldehyde in 1× PBS for 24 h. The tissue was cryopreserved in 20% and 30% sucrose at 4 °C for 24 h, then flash frozen in liquid nitrogen and stored at −80 °C until use. The tissue was cut into 20-μm horizontal sections on a cryostat (CM 1900; Leica).

SC sections were washed three times with 0.01 M PBS and incubated overnight at 4 °C with primary antibody. After three washes with PBS, the sections were incubated with the following primary antibodies: mouse monoclonal anti-GFAP (1:200) to label astrocytes; rabbit and mouse monoclonal anti-nestin (1:200) to label NSCs; and mouse monoclonal anti-Tuj1 (1:200) to label immature neurons. The sections were then incubated for 1 h at room temperature with the following secondary antibodies under protection from light: Alexa Fluor 488 goat anti-rabbit (1:100) and Cy5 goat anti-mouse (1:100). All antibodies were from ABclonal (Woburn, MA, USA). The sections were washed 3 times with PBS and incubated with 4′,6-diamidino-2-phenylindole (DAPI) for 3 min in the dark. Excess DAPI was removed by PBS washes and the specimens were covered with glycerol and a glass coverslip and examined under a fluorescence microscope (model BX-51; Olympus, Tokyo, Japan).

*1.7. Statistical analysis*

All experiments were repeated three times, and data are expressed as mean±SD. Differences between groups were evaluated with the paired Student’s t test using SPSS v23.0 (SPSS, Chicago, IL, USA), with *P*<0.05 considered statistically significant.

[1] Z.Y. Fan, L. Ren, W.J. Zhang, D.D. Li, G.Q. Zhao, J.H. Yu, AIE luminogen-functionalised mesoporous silica nanoparticles as nanotheranostic agents for imaging guided synergetic chemo-/photothermal therapy, Inorganic Chemistry Frontiers 4(5) (2017) 833-839.

[2] S.W. Scheff, D.A. Saucier, M.E. Cain, A statistical method for analyzing rating scale data: the BBB locomotor score, Journal of neurotrauma 19(10) (2002) 1251-60.


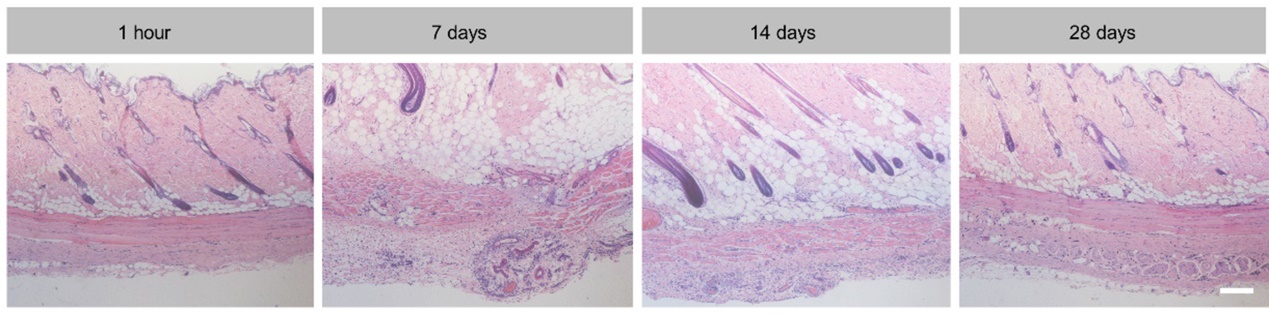


**Figure S1 Biocompatibility of electroactive hydrogel.** Scale bar = 200 μm.
